# Spherical Bi_2_WO_6_/Bi_2_S_3_/MoS_2_ n-p Heterojunction with Excellent Visible-Light Photocatalytic Reduction Cr(VI) Activity

**DOI:** 10.3390/nano10091813

**Published:** 2020-09-11

**Authors:** Jing Ren, Tingting Hu, Qinghua Gong, Qian Wang, Bin Sun, Tingting Gao, Pei Cao, Guowei Zhou

**Affiliations:** Key Laboratory of Fine Chemicals in Universities of Shandong, School of Chemistry and Chemical Engineering, Qilu University of Technology (Shandong Academy of Sciences), Jinan 250353, China; 17862963042@163.com (J.R.); hutingting_1981@163.com (T.H.); 18396814931@163.com (Q.G.); qianwang0510@126.com (Q.W.); ttgao@qlu.edu.cn (T.G.); caopei8956@126.com (P.C.)

**Keywords:** n-p heterojunction, Bi_2_WO_6_/Bi_2_S_3_/MoS_2_, Visible-light, Photocatalytic reduction, Cr(VI)

## Abstract

Exploiting excellent photocatalytic activity and stable heterostructure composites are of critical importance for environmental sustainability. The spherical Bi_2_WO_6_/Bi_2_S_3_/MoS_2_ n-p heterojunction is first prepared via an in situ hydrothermal method using Bi_2_WO_6_, Na_2_MoO_4_·2H_2_O, and CH_4_N_2_S, in which the intermediate phase Bi_2_S_3_ is formed due to chemical coupling interaction of Bi_2_WO_6_ and CH_4_N_2_S. Scanning electron microscopy indicates that the compactness of the sample can be easily adjusted by changing the contents of S and Mo sources in the solution. The results of ultraviolet–visible (UV–vis) diffuse reflectance spectra, photoluminescence, transient photocurrent response, and electrochemical impedance spectra indicate that the formation of heterojunctions contributes to enhancing visible-light utilization and promoting photogenerated carrier separation and transfer. The composite material is used as a catalyst for the visible light photocatalytic reduction of Cr(VI). Remarkably, the optimal Bi_2_WO_6_/Bi_2_S_3_/MoS_2_ n-p heterojunction achieves the greatest Cr(VI) reduction rate of 100% within 75 min (*λ* > 420 nm, pH = 2); this rate is considerably better than the Cr(VI) reduction rate of pure Bi_2_WO_6_. The recycling experiment also reveals that the photocatalytic performance of the n-p heterojunction toward Cr(VI) is still maintained at 80% after three cycles, indicating that the n-p heterojunction has excellent structural stability. The capture experiment proves that the main active species in the system are electrons. The reasonable mechanism of Bi_2_WO_6_/Bi_2_S_3_/MoS_2_ photocatalytic reduction Cr(VI) is proposed. Our work provides new research ideas for the design of ternary heterojunction composites and new strategies for the development of photocatalysts for wastewater treatment.

## 1. Introduction

With the rapid development of industrial production, the pollution of water and land resources by heavy metals is becoming increasingly serious [1,2,3]. Water-soluble, non-biodegradable hexavalent chromium Cr(VI), which easily penetrates through food chain enrichment, can induce cellular oxidative stress, leading to DNA damage, gene mutation, fetal malformation, and carcinogenesis, is one of the most dangerous heavy metals [4,5]. Cr(VI) is widely applied in smelting, electroplating, painting, chemical manufacturing, and tanned leather [6,7,8]. The World Health Organization stipulates that the maximum limit of pollutants in surface water is 0.1 mg L^−1^. However, the concentration of Cr(VI) in sewage is usually higher than 100 mg L^−1^ [9]. Therefore, the development of economical and efficient wastewater chromium removal technology is significant. At present, the methods for reducing the Cr(VI) concentration in aqueous solutions mainly include electrochemical precipitation, ion exchange, membrane filtration, photocatalytic degradation, and adsorption [10,11,12,13,14]. The reduction of Cr(VI) to less toxic Cr(III) by semiconductor photocatalytic technology is considered an effective, low-cost method that generates no harmful substances [15,16,17].

Bismuth-based photocatalysts have always been a research hotspot in the field of photocatalysis. Bismuth tungstate (Bi_2_WO_6_) has been widely studied for its safety, cheap, proper band gap (~2.8 eV), high stability, and excellent photocatalytic activity [18,19,20]. However, bare Bi_2_WO_6_ has the disadvantages of high photo-generated electron-holes recombination efficiency, narrow light absorption range, small specific surface area, and weak surface adsorption capacity, which makes it exhibit poor photocatalytic performance [21,22,23]. Therefore, a scientific strategy that enhances the performance of photocatalysts in practical applications must be developed.

At present, the methods used to improve the photocatalytic activity of semiconductors are as follows: nanostructure modification [24], surface engineering, and homojunction/heterostructure construction. Among these methods, the construction of heterojunctions is considered the most simple and efficient [25,26,27]. The reason is that the establishment of heterojunction cannot only effectively broaden the range of light response and enhance the light absorption of catalyst but also achieve the effective separation of photo-generated carriers under the action of internal electric field and improve the catalytic activity [28,29]. Many Bi_2_WO_6_-based heterojunction photocatalysts, such as Bi_2_WO_6_/MoS_2_ [30], Bi_2_WO_6_/Fe_2_O_3_ [25], Bi_2_WO_6_/Bi_2_S_3_ [31], CdS/Bi/Bi_2_WO_6_ [32], meso-tetra (4-carboxyphenyl) porphyrin/rGO/Bi_2_WO_6_ [33], and Co_3_O_4_/Ag/Bi_2_WO_6_ [34], have been used to improve the performance of pure Bi_2_WO_6_. Huang et al. successfully prepared a new flower-shaped AgBr/Bi_2_WO_6_ catalyst, which showed good catalytic performance in the degradation of tetracycline (TC) under visible light (vis-light) irradiation [35]. Wan et al. prepared Au/Bi_2_WO_6_–MoS_2_ heterojunction photocatalysts, which exhibited excellent vis-light photocatalytic activity in Cr(VI) and tetracycline hydrochloride degradation [23]. Xue et al. synthesized new g-C_3_N_4_/Bi_2_WO_6_/AgI catalyst by a hierarchical assembly method. Compared with bare Bi_2_WO_6_, the ternary heterojunction composite has stronger redox capacity and exhibits better catalytic activity during the photodegradation of organic pollutants such as TC [36]. Long et al. prepared 3D flower-like MoS_2_/Bi_2_S_3_ heterostructures with excellent photocatalytic activity toward the photodegradation of low concentrations of organic pollutants [37]. The above results show that the successful construction of multi-component heterostructure can effectively improve the photocatalytic activity compared with a single component. However, the Bi_2_WO_6_-based photocatalysts still have disadvantages, including a complex preparation method, narrow light response range, and rapid photogenerated charge carrier recombination. In recent years, the use of MoS_2_ and Bi_2_S_3_ coupled with other semiconductors for boosted photocatalytic performance has been widely investigated. To our knowledge, the preparation and application in photocatalysis of the Bi_2_WO_6_/Bi_2_S_3_/MoS_2_ ternary heterojunction has not been reported. Hence, seeking a facile and controllable preparation method to fabricate ternary heterojunctions containing Bi_2_WO_6_, Bi_2_S_3_, and MoS_2_ is of great importance for improving the photocatalytic performance of Bi_2_WO_6_ in environmental purification.

**Scheme 1:**
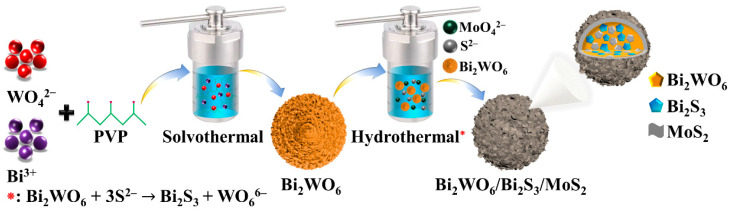
Schematic diagram of preparing Bi_2_WO_6_/Bi_2_S_3_/MoS_2_ heterojunction ternary composites.

Based on the above considerations, in the present work, Bi_2_WO_6_/Bi_2_S_3_/MoS_2_ heterojunction ternary composite materials are prepared via hydrothermal method using the synthesized Bi_2_WO_6_ microspheres as substrate (Scheme 1) and which are used for photocatalytic reduction of Cr(VI) to Cr(III). Importantly, the formation of Bi_2_S_3_ does not require an additional Bi source, and S^2−^ partially replaces WO_6_^6−^ in Bi_2_WO_6_. This process maintains a superior spherical structure and is beneficial to the uniform distribution of Bi_2_S_3_ in the composite. The compactness of composite nanoflakes can be easily adjusted by changing the content of sodium molybdate dihydrate (Na_2_MoO_4_·2H_2_O) and thiourea (CH_4_N_2_S) in the solution during the hydrothermal process. To the best of our knowledge, the Bi_2_WO_6_/Bi_2_S_3_/MoS_2_ heterojunction for Cr(VI) photocatalytic reduction under vis-light irradiation is investigated for the first time. The composites have higher adsorption capacity and photocatalytic activity than pure Bi_2_WO_6_ in terms of Cr(VI) reduction due to the successful construction of heterojunction structure. The synergistic effect among the three components enhances light absorption and realizes the effective separation and transmission of photogenerated carriers. The Cr(VI) reduction rate of Bi_2_WO_6_/Bi_2_S_3_/MoS_2_ reaches 100% within 75 min (*λ* > 420 nm, pH = 2) and is considerably better than that of the pure Bi_2_WO_6_. These results provide new research ideas for the design of ternary heterojunctions to develop highly efficient vis-light-driven photocatalysts for wastewater treatment.

## 2. Experimental Section

### 2.1. Materials and Chemicals

All chemicals and materials in this work were of analytical grade and purchased from commercial suppliers, which could be directly utilized without any further purification. Sodium tungstate dihydrate (Na_2_WO_4_·2H_2_O, AR, 99.5%), Na_2_MoO_4_·2H_2_O (AR, 99.0%), bismuth(III) nitrate pentahydrate (Bi(NO_3_)_3_·5H_2_O, AR, 99.0%), and CH_4_N_2_S (AR, 99.0%) were acquired from Sigma-Aldrich (St. Louis, MO, USA). Absolute ethanol (C_2_H_5_OH, AR, ≥99.7%), glacial acetic acid (CH_3_COOH, AR, ≥99.5%), polyvinyl pyrrolidone K30 (PVP K30, AR), and other chemicals used in the experiments were bought from Shanghai Chemical Reagent Co., Ltd. (Shanghai, China). Ultrapure water (18.2 MΩ cm^−1^) was served throughout the study and acquired from the Milli-Q water purifying system (Millipore Corp., Bedford, MA, USA).

### 2.2. Synthesis of Spherical Bi_2_WO_6_ Nanostructures

Solutions A and B were prepared in the synthesis of Bi_2_WO_6_ precursor. In solution A, 2 mmol Bi(NO_3_)_3_·5H_2_O and 4 g PVP K30 were added to a mixed solution of 50 mL ultrapure water, absolute ethanol, and glacial acetic acid with a 3:1:1 volume ratio and then stirred at room temperature for 60 min. In solution B, 1 mmol Na_2_WO_4_·2H_2_O was added to 20 mL H_2_O for 30 min of ultrasonication. After the solutions were clarified, solution B was dropped to solution A under agitation and stirred continuously for 60 min to obtain a white uniform suspension. The suspension was transferred to a Teflon-sealed autoclave (100 mL) for a solvothermal reaction at 180 °C for 18 h. After cooling, the light-yellow product was collected, washed thrice with absolute ethanol and ultrapure water in sequence, and finally dried overnight and ground for reserves.

### 2.3. Synthesis of Bi_2_WO_6_/Bi_2_S_3_/MoS_2_ n-p Heterojunction Photocatalyst

Bi_2_WO_6_/Bi_2_S_3_/MoS_2_ n-p heterojunction nanocomposites were prepared by a simple hydrothermal reaction. First, 200 mg Bi_2_WO_6_ was dispersed in 40 mL water with ultrasonic treatment for 10 min. Further 1 h stirring treatment was needed after the addition of 200 mg Na_2_MoO_4_·2H_2_O and 400 mg CH_4_N_2_S as an ion source. Second, the dispersion was transferred to 100 mL Teflon-sealed autoclave for hydrothermal reaction at 200 °C for 24 h. Wait for cooling, the obtained sample (named as product BBM-3) was rinsed with ultrapure water and anhydrous ethanol for thrice and then dried overnight at 60 °C in a vacuum oven. As a control, the addition amount of Na_2_MoO_4_·2H_2_O:CH_4_N_2_S was adjusted (80 mg:160 mg, 120 mg:240 mg, 300 mg:600 mg), and the corresponding products with BBM-1, BBM-2, and BBM-4 were expressed. In addition, the preparation method of pure MoS_2_ nanosheets was similar to the above process except that Bi_2_WO_6_ was not added.

### 2.4. Characterization

Powder X-ray diffraction (XRD) patterns were obtained on a SmartLab SE X-ray diffractometer (Rigaku Corp., Tokyo, Japan) with Cu Kα (*λ* = 1.5046 Å) radiation. Raman spectra were measured using a Renishaw in Via9 Raman microscope system (Renishaw, London, UK) with a 50× objective and a 532 nm laser irradiation to focal point the laser beam into a spot with a diameter of approximately 1 µm. The morphology and energy-dispersive spectra (EDS) of the samples were tested and characterized by a field-emission scanning electron microscope (FESEM, Regulus 8220, Hitachi, Tokyo, Japan). The microstructure and lattice fringe of the samples were examined by a transmission electron microscope (TEM, JEM-2100, JEOL, Tokyo, Japan) and high-resolution TEM (HRTEM, JEM-2100). The elemental composition and chemical state of the samples were determined by X-ray photoelectron spectroscopy (XPS) (Perkin-Elmer PHI5300 spectrometer, Perkin Elmer, Waltham, MA, USA). The specific surface areas of the samples were obtained based on the N_2_ adsorption–desorption isotherm tested on a Micromeritics ASAP 2460 system (ASAP, Norcross, GA, USA). The ultraviolet–visible (UV–vis) diffuse reflectance spectra (DRS) were tested within a 200 nm to 800 nm wavelength range using a spectrometer (UV-2600, Shimadzu, Kyoto, Japan) with BaSO_4_ as a reference. A Hitachi F4500 fluorescence spectrophotometer (Hitachi, Tokyo, Japan) was used to test the photoluminescence (PL) measurements (*λ*_excitation_ = 300 nm). The Mott–Schottky curves, electrochemical impedance spectra (EIS), and photocurrent response experiments were carried out using the electrochemical workstation (PARSTAT 4000, Ametec, Berwyn, PA, USA) with a conventional three-electrode configuration (working electrode: fluorine-doped tin oxide conducting glass; counter electrode: platinum plate; reference electrode: Ag/AgCl electrode) and Na_2_SO_4_ aqueous solution as the electrolyte (0.1 mol L^−1^).

### 2.5. Photocatalytic Activity Experiments

The specific operational steps of the prefabricated catalysts for photocatalytic reduction of Cr(VI) (Cr(VI) source: K_2_Cr_2_O_7_) were as follows: Cr(VI) solution with concentration of 40 mg L^−1^ was prepared using ultrapure water as solvent. Then, 50 mL of this initial solution was accurately measured and placed in the reaction vessel. Next, the initial solution pH to 2 was adjusted with HCl solution (1 mol L^−1^). Afterward, the 20 mg as-synthesized catalysts were evenly dispersed in the solution by ultrasonication. Before vis-light irradiation, the suspension was stored in the dark place and stirred for 60 min to reach equilibrium of adsorption and desorption. Under the irradiation by Xe lamp (300 W, 100 mW cm^−2^, *λ* > 420 nm), 3 mL suspension was taken out in the reaction container every 15 min and centrifuged (9000 r min^−1^, 10 min). Then, the supernatant was collected with microporous (0.22 μm) membrane filter syringe to eliminate residual particles. NaOH (1 mol L^−1^) or HCl (1 mol L^−1^) solution was used to adjust the pH of Cr(VI) solution to investigate the effect of solution pH on photocatalysis. Finally, the Cr(VI) concentration was obtained by measuring the absorbance of the supernatant at 540 nm (UV-2600, Shimadzu) with diphenylcarbazide approach (Electronic Supplementary Materials).

## 3. Results and Discussion

### 3.1. X-ray Diffraction (XRD) and Raman Analysis

**Figure 1:**
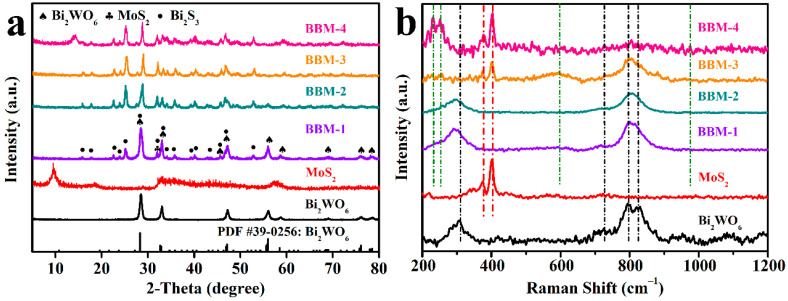
X-ray diffraction (XRD) patterns (**a**), and Raman spectra (**b**) of bare Bi_2_WO_6_, MoS_2_, BBM-1, BBM-2, BBM-3, and BBM-4 heterojunction.

Figure 1a shows the XRD patterns of the products. For Bi_2_WO_6_, the diffraction peaks at 2*θ* = 28.3°, 32.8°, 47.1°, 56.0°, 58.5°, 68.8°, 76.1°, and 78.5° correspond to the (131), (200), (202), (133), (262), (400), (2102), and (204) crystal faces of the Bi_2_WO_6_ orthorhombic phase (JCPDS Card No.39-0256), respectively [38,39]. The three diffraction peaks of bare MoS_2_ at 9.0°, 32.0°, and 58.0° correspond to the (002), (100), and (110) crystal faces of 2H-MoS_2_, respectively. The peaks at 9.0° and 17.0° indicate the formation of a layered structure with enlarged interlayer spacing [40]. For heterojunction photocatalysts, the XRD patterns display new diffraction peaks. The diffraction peaks located at 25.0° correspond to the (130) crystal plane of Bi_2_S_3_ [41]. Given the strong interaction force between Bi^3+^ and S^2−^, Bi_2_S_3_ will be formed at relatively high temperatures [42]. The Bi_2_WO_6_/Bi_2_S_3_/MoS_2_ samples show a discernible peak at approximately 32.0°, which is attributed to the (100) crystal plane of MoS_2_, indicating that the composite material contains MoS_2_ component. However, the absence of the highest MoS_2_ intensity peak (~9.0°) from the heterostructure samples indicates that MoS_2_ nanosheets may contain only a few layers that are too thin to be detected by XRD [43].

Raman measurements of the as-synthesized samples were performed in the range of 200–1200 cm^−1^, and the results are shown in Figure 1b. The black, green, and red dashed lines in the figure represent the Raman characteristic peaks of Bi_2_WO_6_, Bi_2_S_3_, and MoS_2_, respectively. The peaks of 308, 723, 798, and 829 cm^−1^ in the Raman spectra are characteristic Raman shifts of Bi_2_WO_6_ [44]. The Raman peaks of Bi_2_S_3_ are located at 234.8, 260, 590, and 970 cm^−1^, of which the peaks at 234.8 and 260 cm^−1^ matched the A_g_^1^ and B_1g_ vibration mode, respectively [45]. Meanwhile, the typical peaks at 383 and 408 cm^−1^ are ascribed to the E^1^_2g_ and A_1g_ vibrations of MoS_2_, respectively [42,45]. Based on the above results, the Bi_2_WO_6_/Bi_2_S_3_/MoS_2_ ternary composites are successfully prepared.

### 3.2. Morphology

**Figure 2:**
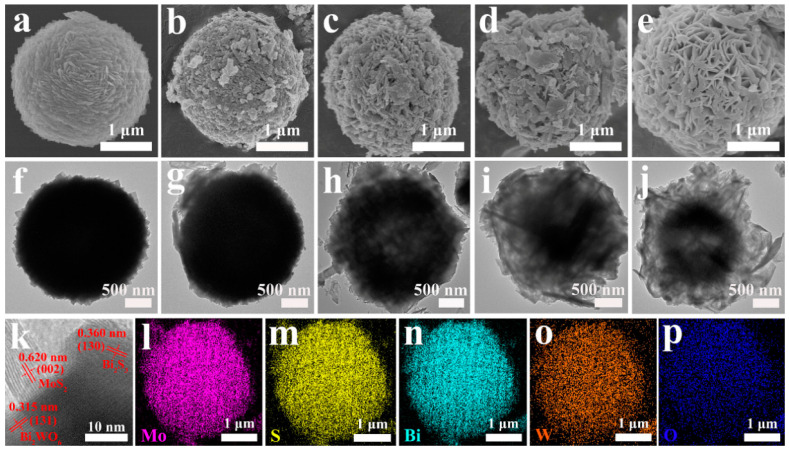
Scanning electron microscope (SEM) and transmission electron microscope (TEM) images of (**a**,**f**) bare Bi_2_WO_6_, (**b**,**g**) BBM-1, (**c**,**h**) BBM-2, (**d**,**i**) BBM-3, and (**e**,**j**) BBM-4. High-resolution TEM (HRTEM) image (**k**), and energy-dispersive spectra (EDS) mapping images (**l**–**p**) of BBM-3.

As shown in the scanning electron microscope (SEM) images (Figure 2a), Bi_2_WO_6_ microspheres with diameters of 2.6–3.0 μm are self-assembled from nanosheets. The SEM images in Figure 2b–e show that the degree of looseness of the Bi_2_WO_6_/Bi_2_S_3_/MoS_2_ microsphere increases accordingly with the increase in Mo and S sources concentration during the hydrothermal process. Nevertheless, with the further increase in concentration, MoS_2_ agglomerates are formed on the Bi_2_WO_6_/Bi_2_S_3_/MoS_2_ surface, and the corresponding results are shown in Figure 2e. This finding is consistent with the information expressed in the TEM diagram in Figure 2f–j. The causes of these phenomenon are as follows: I. A strong affinity exists between Bi^3+^ and S^2−^, which reacts under high temperature and pressure to form Bi_2_S_3_ (Bi_2_WO_6_ + 3S^2−^ → Bi_2_S_3_ + WO_6_^6−^) [42,45]. II. Bi_2_WO_6_ is consumed during this process, and Bi_2_S_3_ and MoS_2_ are generated. As the consumption of Bi_2_WO_6_ increases, the structure becomes looser. III. The formation of MoS_2_ agglomerates is mainly caused by the excessively high concentration of Mo and S sources, which promotes the nucleation speed to extreme degrees [46]. Soon afterward, BBM-3 is used as the model, and its composition is characterized by high-resolution transmission electron microscopy (HRTEM). Figure 2k shows the tight interface between Bi_2_WO_6_, Bi_2_S_3_, and MoS_2_ in the BBM-3. The measured interplanar distances of 0.315, 0.360, and 0.620 nm belong to the (131) crystal plane of orthorhombic Bi_2_WO_6_, the (130) plane of Bi_2_S_3_, and the (002) lattice plane of MoS_2_, respectively [47,48,49]. The high-magnification TEM of BBM-3 (Figure S1), which can intuitively illustrate the close interface contact between Bi_2_WO_6_, Bi_2_S_3_, and MoS_2_, confirms the successful construction of the Bi_2_WO_6_/Bi_2_S_3_/MoS_2_ heterojunction [42]. Meanwhile, element mapping is used to analyze BBM-3 in depth to further determine the distribution of Mo, S, Bi, W, and O in the material. The results (Figure 2l–p) coincide with the EDS characterization results (Figure S2), confirming that BBM-3 consists of Mo, S, Bi, W, and O. The above results confirm that Bi_2_WO_6_/Bi_2_S_3_/MoS_2_ n-p heterojunction with spherical structure can be synthesized by a simple in-situ hydrothermal method.

### 3.3. X-ray Photoelectron Spectroscopy (XPS) Analysis

**Figure 3:**
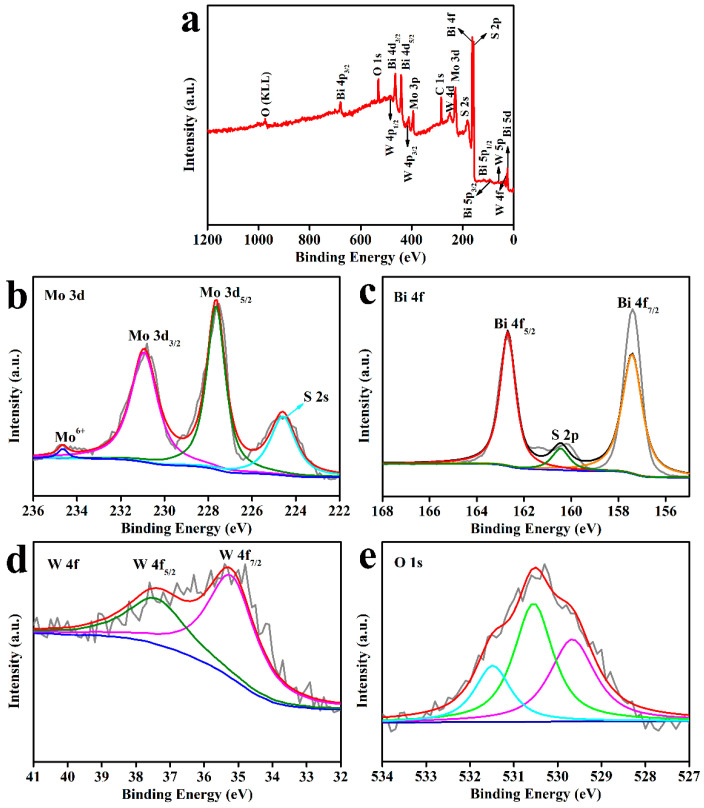
X-ray photoelectron spectroscopy (XPS) analysis of BBM-3 showing (**a**) survey, (**b**) Mo 3d, (**c**) Bi 4f, (**d**) W 4f, and (**e**) O 1s spectra.

The survey XPS curves in Figure 3a indicate that BBM-3 is composed of Mo, Bi, S, W, and O. Figure 3b–e shows the high-resolution spectra of Mo 3d, Bi 4f, W 4f, and O1s, respectively. The Mo 3d (Figure 3b) shows two peaks centered at 227.6 and 230.8 eV, which correspond to Mo 3d_5/2_ and Mo 3d_3/2_ of Mo^4+^, respectively [50]. The satellite peak at approximately 234.7 eV represents Mo^6+^ [51]. In addition, the mid-strong peak at 225.4 eV can be well matched to S 2s [50]. The characteristic signal in Bi 4f diagram (Figure 3c) is formed by Bi 4f_7/2_ at 157.4 eV, Bi 4f_5/2_ at 162.7 eV, and S 2p at 160.6 eV [52]. The difference between the binding energy of Bi 4f_7/2_ and Bi 4f_5/2_ is 5.3 eV, indicating that Bi exists in BBM-3 as Bi^3+^. Figure 3d contains the peaks at 35.2 and 37.6 eV, which are characteristics of W 4f_7/2_ and W 4f_5/2_, respectively [53]. The three fitted peaks in the O 1s spectrum (Figure 3e) are located at 531.5, 530.6, and 529.7 eV, which indicates that three types of O are present in BBM-3. The peak at 531.5 eV represents the chemically adsorbed oxygen (O–H) on the surface of BBM-3, whereas the peaks at 530.6 and 529.7 eV correspond to the O–Bi and O–W lattice oxygen in BBM-3, respectively [54]. Compared with the peaks of pure Bi_2_WO_6_, Bi_2_S_3_, and MoS_2_, the Mo 3d and Bi 4f peaks of BBM-3 display a shift ~1.0 eV to the lower binding energy direction. Conversely, the W 4f peak of BBM-3 shows a shift ~1.0 eV to the higher binding energy direction. These results are primarily due to strong interactions and charge transfer among Bi_2_WO_6_, Bi_2_S_3_, and MoS_2_ in BBM-3 (Figure S3) [29]. The above XPS analyses confirm again that Bi_2_WO_6_, Bi_2_S_3_, and MoS_2_ coexist in the Bi_2_WO_6_/Bi_2_S_3_/MoS_2_ ternary heterojunction photocatalyst.

### 3.4. Brunauer–Emmett–Teller (BET) Specific Surface Area Analysis

As shown in Figure S4, the Bi_2_WO_6_ and Bi_2_WO_6_/Bi_2_S_3_/MoS_2_ ternary heterojunction samples exhibit type IV isotherms, which indicate the existence of mesoporous structures [11,22]. The Brunauer–Emmett–Teller (BET) surface area of Bi_2_WO_6_/Bi_2_S_3_/MoS_2_ composites is higher than that of bare Bi_2_WO_6_ (14.7 m^2^ g^−1^). The BET surface areas of BBM-1, BBM-2, BBM-3, and BBM-4 are 16.6, 19.7, 22.7, and 19.4 m^2^ g^−1^, respectively. Compared with pure Bi_2_WO_6_, Bi_2_WO_6_/Bi_2_S_3_/MoS_2_ composites have high BET surface areas and rich mesoporous structures, which facilitate the adsorption and reduction of Cr(VI).

### 3.5. Ultraviolet–Visible (UV–Vis) Absorption and Band Gap Positions

**Figure 4:**
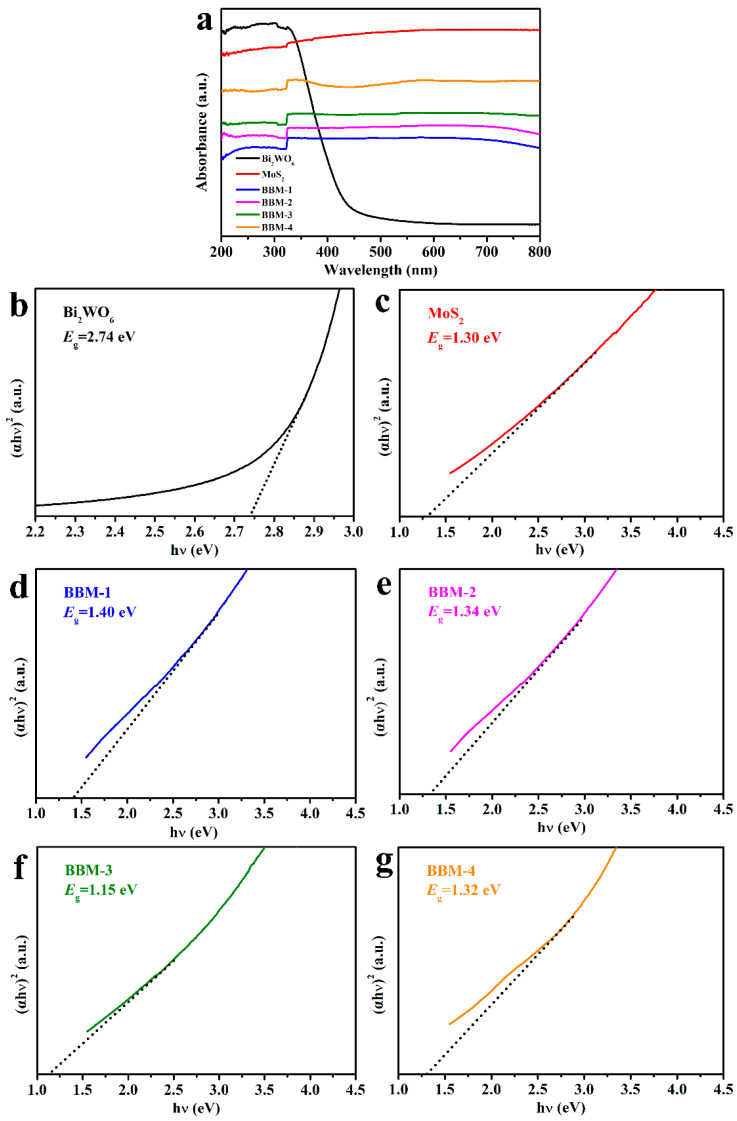
(**a**) Ultraviolet–visible (UV–vis) diffuse reflectance spectra (DRS) spectra of Bi_2_WO_6_, MoS_2_, BBM-1, BBM-2, BBM-3, and BBM-4. (**b**–**g**) Plot of (*αhν*)^2^ versus photon energy of Bi_2_WO_6_, MoS_2_, BBM-1, BBM-2, BBM-3, and BBM-4.

The DRS of the pristine Bi_2_WO_6_, pristine MoS_2_, and ternary composites are recorded to investigate the light absorption of the samples. As shown in Figure 4a, for the pristine Bi_2_WO_6_, its intrinsic light absorption edge is at 450 nm, which means that the material has light absorption only in the UV and partially visible regions. By contrast, pure MoS_2_ shows a strong absorption in the UV–vis region. As expected, Bi_2_WO_6_/Bi_2_S_3_/MoS_2_ ternary heterojunction photocatalyst extends the vis-light absorption range compared with the Bi_2_WO_6_. Thus, the good photocatalysis performance of the composite is predicted. Furthermore, the bandgap energy (*E*_g_) of as-fabricated materials is obtained in accordance with Tauc’s equation (Equation (1)) [44,55]:

(1) $$(\alpha h\nu )\;=\;A(h\nu \;-{\;E}_{g}{)}^{n/2},$$

where *α*: absorption coefficient, *h*: Planck’s constant, *ν*: light frequency, and A: a constant.

The value of *n* depends on the type of electronic transition in the semiconductor (*n* values of direct/indirect transition: 1/4). According to the previous reports, the *n* of Bi_2_WO_6_ and MoS_2_ is 1, and their *E*_g_ are determined by the extrapolation of Tauc linear region [28,56]. The *E*_g_ of pure Bi_2_WO_6_ and MoS_2_ are ~2.74 and 1.30 eV, respectively (Figure 4b,c), which are close to previously reported values [30,36]. The *E*_g_ of BBM-1, BBM-2, BBM-3, and BBM-4 composites are ~1.40, 1.34, 1.15, and 1.32 eV, respectively (Figure 4d–g).

On the above basis, the valence band (VB) and conduction band (CB) edge potentials of the samples are calculated in accordance with the Mulliken atomic electronegativity theory (Equations (2) and (3), respectively) [30,51]:

(2) $$E_{\mathrm{CB}}\;+\;0.5E_{g}\;=\;X\;-{\;E}^{e},$$

(3) $$E_{\mathrm{VB}}{\;=\;E}_{\mathrm{CB}}{\;+\;E}_{g},$$

where *E*_g_ and *X* represent the band gap energy and absolute electronegativity, respectively. *E*_VB_ and *E*_CB_ are the VB and CB edge, respectively. *E*^e^ is energy of free electrons (~4.5 eV) on the hydrogen scale. Table 1 shows the calculation results of material-related parameters. The flat-band potentials of the related materials are studied by using Mott–Schottky curves (Figure S5) to verify the rationality of the calculation results. As presented in Figure S5a–c, Bi_2_WO_6_ is classified as an n-type semiconductor due to its positive slope, whereas Bi_2_S_3_ and MoS_2_ are confirmed as p-type semiconductors due to their negative slopes. When they are coupled to each other to form a n-p heterojunction (Bi_2_WO_6_/Bi_2_S_3_/MoS_2_), the Mott–Schottky curve shows an inverted ‘V-shape’ characteristic (Figure S5d). Generally, *E*_VB_ for p-type semiconductors is very close to the flat-band potential, whereas *E*_CB_ for n-type semiconductors is very close to the flat-band potential [57]. The flat-band potential in the n-type semiconductor is 0.1–0.3 eV higher than *E*_CB_, whereas that in the p-type semiconductor is 0.1–0.3 eV lower than *E*_VB_ [58]. Figure S5a–c shows that the flat-band potentials of pure Bi_2_WO_6_, Bi_2_S_3_, and MoS_2_ can be confirmed to be 0.20 (0.40 eV vs. normal hydrogen electrode (NHE)), 1.06 (1.26 eV vs. NHE), and 1.14 (1.34 eV vs. NHE), respectively. Therefore, *E*_CB_ of pure Bi_2_WO_6_ and *E*_VB_ of Bi_2_S_3_ and MoS_2_ can be estimated to be 0.33, 1.37, and 1.47 eV. On the basis of Equation (3), the corresponding *E*_VB_ of Bi_2_WO_6_ and *E*_CB_ of Bi_2_S_3_ and MoS_2_ can occur at approximately 3.07, 0.18, and 0.17 eV. These results are in agreement with the result calculated in accordance with Mulliken atomic electronegativity theory.

### 3.6. Photoelectrochemical Performance

**Figure 5:**
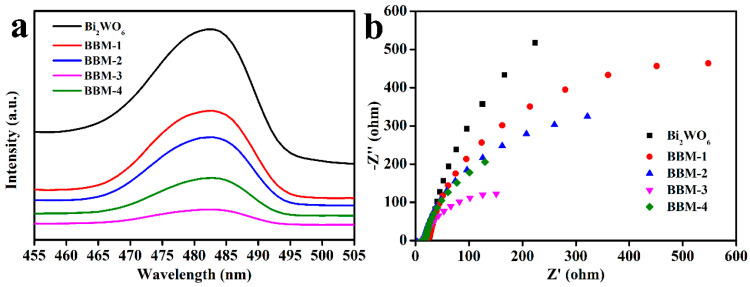
(**a**) Photoluminescence spectra (*λ*_excitation_ = 300 nm) and (**b**) electrochemical impedance spectra (EIS) spectra of the Bi_2_WO_6_, BBM-1, BBM-2, BBM-3, and BBM-4.

The catalyst is further characterized by PL spectroscopy and photocurrent response to explore its charge separation efficiency. High PL intensity indicates low charge separation efficiency and easy electron-hole recombination, whereas the photocurrent response shows the opposite [35,59,60]. The PL spectra (Figure 5a) of Bi_2_WO_6_ and Bi_2_WO_6_/Bi_2_S_3_/MoS_2_ composites are obtained under the condition of *λ*_excitation_ = 300 nm. The emission intensities of all Bi_2_WO_6_/Bi_2_S_3_/MoS_2_ composites are significantly lower than that of bare Bi_2_WO_6_. Based on the intensity, the composites can be sorted as Bi_2_WO_6_ > BBM-1 > BBM-2 > BBM-4 > BBM-3. This result shows that the successful recombination of MoS_2_, Bi_2_S_3_, and Bi_2_WO_6_ improves the efficiency of charge separation. The photocurrent response (Figure S6) confirms this conclusion. In the experimental process of up to 400 s, BBM-3 consistently shows the highest photocurrent. The EIS test can be used to explore the interface charge transfer properties, with the small arc radius reflecting a fast charge transfer speed [61]. Figure 5b shows the Nyquist plots of Bi_2_WO_6_ and composites. The composites exhibit a smaller Nyquist plot semicircle radius compared with pure Bi_2_WO_6_. BBM-3 also shows a considerably smaller semicircle radius of EIS Nyquist plots than the other composites (BBM-1, BBM-2, and BBM-4), which is highly consistent with the PL and photocurrent test analysis results. Therefore, the following conclusions can be drawn. First, the construction of Bi_2_WO_6_, Bi_2_S_3_, and MoS_2_ heterostructures can significantly improve the charge separation efficiency. Second, only when Bi_2_WO_6_ is compounded with suitable amount of Bi_2_S_3_ and MoS_2_ can n-p heterojunction photocatalysts be formed effectively. Thus, extremely high and extremely low compounding ratios are not conducive to the formation of heterostructures and the separation and transfer of photogenerated carriers. Third, BBM-3 is expected to have the best vis-light catalytic activity because it enables the effective separation of photo-generated carriers.

### 3.7. Photocatalytic Activity

**Figure 6:**
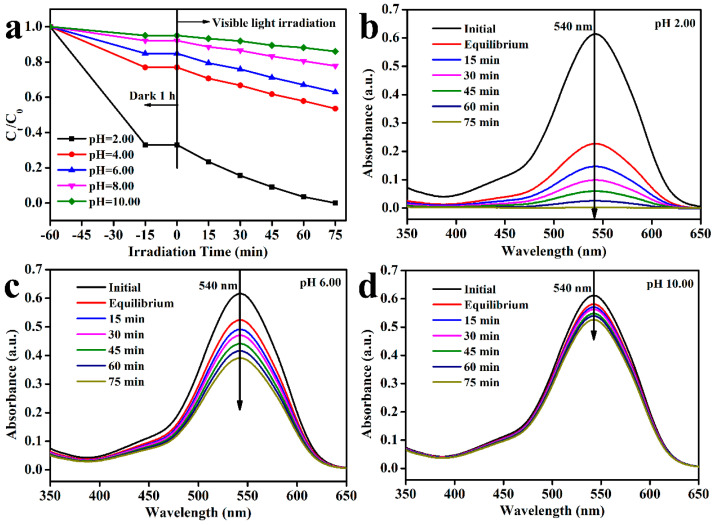
(**a**) Effect of pH on the catalytic activity of BBM-3. At the pH of 2.00, 6.00, and 10.00 (**b**–**d**, respectively), the UV–vis absorption spectra of Cr(VI) solution on BBM-3 sample changed.

In this working system, the catalytic reduction of Cr(VI) under vis-light irradiation is used as the evaluation standard to evaluate the performance of the prefabricated materials. Previous reports have shown that the initial solution pH strongly influences the photocatalytic Cr(VI) reduction. Therefore, the pH of the initial solution is adjusted to show a linear gradient change, which is used to investigate the effect of pH on the catalytic activity of BBM-3. The photocatalytic reduction efficiency of BBM-3 is the highest under acidic conditions (Figure 6a). Under the condition of pH = 2.00, the Cr(VI) reduction rate of BBM-3 is as high as 100%. With the increase in pH, the reduction rate of Cr(VI) shows a strictly decreasing trend. When pH = 10.00, the reduction efficiency of Cr(VI) reaches 14%. This change is confirmed by the corresponding UV–vis absorption spectra (Figure 6b–d and Figure S7). The above situation is mainly caused by the following factors. First, Cr(VI) mainly exists in the form of HCrO_4_^−^ and Cr_2_O_7_^2−^ in acidic environments and CrO_4_^2−^ in alkaline environments [62]. When the solution environment is strongly acidic, the hydroxyl groups on the surface of the catalyst will be protonated to become (–OH_2_^+^), which in turn enhances the electrostatic adsorption on Cr(VI) [61]. Second, the reactions in acidic conditions are as follows (Equations (4) and (5)) [17,61,63]:

(4) $${\mathrm{HCrO}}_{4}^{-}{\;+\;7H}^{+}{\;+\;3e}^{-}\to {\;\mathrm{Cr}}^{3+}{\;+\;4H}_{2}O,$$

(5) $${\mathrm{Cr}}_{2}O_{7}^{2-}{\;+\;14H}^{+}{\;+\;6e}^{-}\;\to {\;2\mathrm{Cr}}^{3+}{\;+\;7H}_{2}O,$$

The reaction under alkaline conditions is as follows (Equation (6)) [17,61,63]:

(6) $${\mathrm{CrO}}_{4}^{2-}{\;+\;4H}_{2}{O\;+3e}^{-}\;\to {\;\mathrm{Cr}(\mathrm{OH})}_{3}{\;+\;5\mathrm{OH}}^{-},$$

Equation (6) shows that Cr^3+^ will be converted into Cr(OH)_3_ and deposited on the catalyst surface under alkaline conditions, blocking the active sites that can be used for adsorption and photocatalytic reactions. In summary, acidic conditions are more conducive to the reduction of Cr(VI) than alkaline conditions.

**Figure 7:**
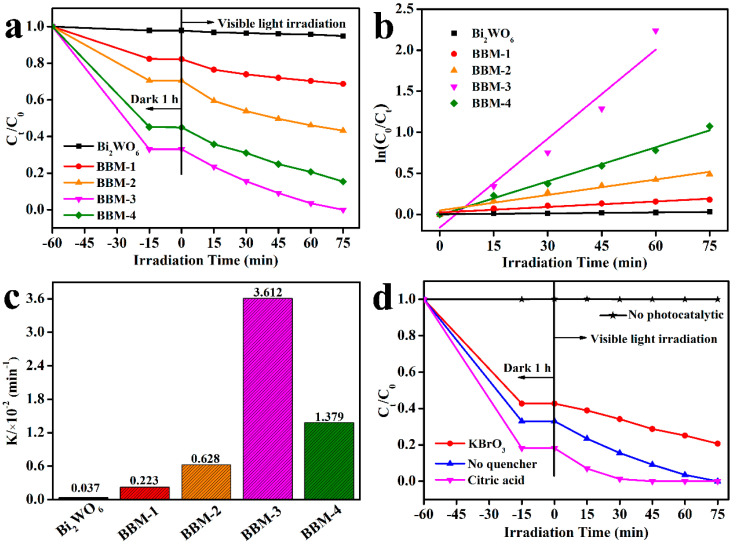
(**a**) Visible light (vis-light) catalytic reduction of Cr(VI) by different catalysts. (**b**) Corresponding pseudo first-order kinetic curves, and (**c**) rate constant “k” by different catalysts. (**d**) BBM-3 vis-light catalytic reduction Cr(VI) in the presence of electron scavengers (KBrO_3_) and hole scavengers (citric acid), respectively. Dosages of KBrO_3_ and citric acid aqueous solutions: 100 μL of 50 mg mL^−1^.

The photocatalytic activities of Bi_2_WO_6_ and different Bi_2_WO_6_/Bi_2_S_3_/MoS_2_ composites are evaluated at the initial solution pH = 2.00, and the results are shown in Figure 7a. The composites exhibit high Cr(VI) adsorption performance compared with pure Bi_2_WO_6_, which benefits from the high surface area and abundant mesoporous structure of the composite. After 75 min of irradiation, the reduction rate of Bi_2_WO_6_ to Cr(VI) is 5%. By contrast, all the Bi_2_WO_6_/Bi_2_S_3_/MoS_2_ composites manifest remarkably high photocatalytic reduction activity under the same conditions. In particular, BBM-3 shows the best photocatalytic performance with a corresponding Cr(VI) reduction rate of up to 100%. It is not difficult to find that there is an optimal compounding ratio between Bi_2_S_3_, MoS_2_, and Bi_2_WO_6_ occurs in the removal of Cr(VI). Extremely low or extremely high compounding ratio is not conducive to enhancing the photoreduction activity of Bi_2_WO_6_/Bi_2_S_3_/MoS_2_. When the actual ratio is lower than the optimal ratio, the number of active sites used to capture carriers increases with the increase in recombination ratio, thus prolonging the carrier lifetime and then increasing the photocatalytic activity. However, when the compounding ratio is higher than the optimum, excess MoS_2_ will agglomerate to disrupt the effective construction of n-p heterojunction, as confirmed by the SEM result (Figure 2e).

Figure 7b shows the pseudo first-order kinetic curves of Bi_2_WO_6_, BBM-1, BBM-2, BBM-3, and BBM-4 for the photocatalytic reduction of Cr(VI) and the apparent reaction rate constant “*k*” (Figure 7c). The pseudo first-order model demonstrated here is shown by Equation (7) [41]:

(7) $$ln\left(\frac{C_{0}}{C_{t}}\right)\;=\;kt,$$

where *t* stands for the vis-light exposure time, *C*_0_ represents the original concentration of Cr(VI) solution, and *C*_t_ is the concentration of Cr(VI) solution at “*t*” irradiation time. The “*k*” values calculated by the linear fit of *ln*(*C*_0_/*C*_t_) and irradiation time (min) plots are 0.037, 0.223, 0.628, 3.612, and 1.379 × 10^−2^ min^−1^ for Bi_2_WO_6_, BBM-1, BBM-2, BBM-3, and BBM-4, respectively. BBM-3 obtains the highest *k* value. Immediately afterwards, an active species capture experiment is carried out to further explore the mechanism involved in the reaction system (hole/electron trapping agent: citric acid/KBrO_3_). The obtained results are shown in Figure 7d. First, the Cr(VI) solution without photocatalyst shows good stability under vis-light exposure. However, KBrO_3_ significantly inhibited the photoreduction of Cr(VI) by BBM-3 with a final reduction rate of 75%. This finding indicates that the main active material in the catalytic reduction of Cr(VI) process is photogenerated electrons, which is consistent with previous reports [64]. By contrast, with the addition of citric acid, the adsorption and reduction rate of Cr(VI) by BBM-3 are significantly improved. The factors that cause this phenomenon are as follows: First, the surface of the catalyst becomes more positive by the addition of citric acid, which promotes the adsorption of HCrO_4_^−^ or Cr_2_O_7_^2−^ ions [65]. Second, photogenerated holes can oxidize citric acid, which is equivalent to promoting the separation of photogenerated carriers and prolonging the lifetime of photogenerated electrons [61,66]. The above conclusion is confirmed by the corresponding UV–vis absorption spectra in Figure S8. Finally, compared with the other reported catalysts for vis-light reduction of Cr(VI) (Table 2), Bi_2_WO_6_/Bi_2_S_3_/MoS_2_ heterojunction composites show relatively satisfactory photocatalytic activity.

**Figure 8:**
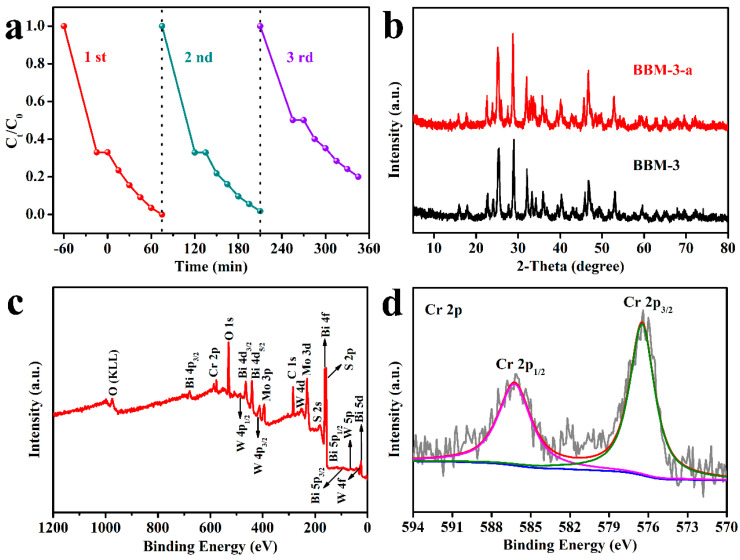
(**a**) Vis-light catalytic reduction of Cr(VI) by BBM-3 at different recycling runs. (**b**) XRD pattern of the BBM-3 and BBM-3-a. XPS spectra of BBM-3-a: (**c**) survey and (**d**) Cr 2p.

Issues such as photocatalysts separation, recovery, reuse, and stability, are important in practical applications. The stability and reusability of BBM-3 after the reaction is proven by recycling and reusing the same catalyst for three cycles. The photoreduction rate of Cr(VI) after three cycle tests is 80% (Figure 8a), which shows that the photocatalysts have enough stability and reusability. This is mainly attributed to the existence of electrostatic attraction that induces a strengthened coupling interaction among Bi_2_WO_6_, Bi_2_S_3_, and MoS_2_; this interaction is beneficial to improving structural stability [51,52]. Figure S9 shows the UV–vis absorption spectra after the second and third cycles. Furthermore, we collected composite samples after use in three photocatalytic cycles (BBM-3-a) and further characterized them by XRD and XPS. The positions of the characteristic peaks of the samples after circulation (Figure 8b) exhibit no change compared with the initial sample. The evident signals of Bi, W, S, Mo, O, and Cr contaminants can be observed in the survey XPS curves of BBM-3-a (Figure 8c). The XPS peak of 577.1 eV in the Cr 2p spectrum (Figure 8d) belongs to Cr 2p_3/2_, which highly matches Cr(III) in Cr(OH)_3_ [73]. To sum up, Bi_2_WO_6_/Bi_2_S_3_/MoS_2_ heterojunction composite has high structural stability, good reusability, and can effectively reduce the toxicity of Cr(VI) to reduce it to Cr(III).

### 3.8. Possible Photocatalytic Mechanism

**Scheme 2:**
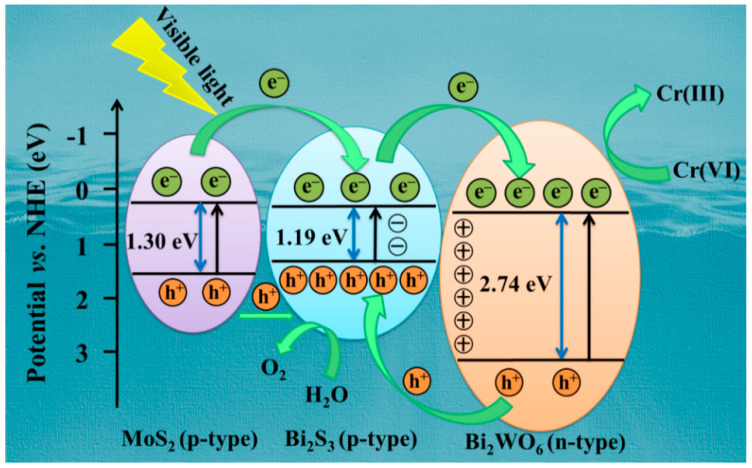
Proposed photocatalytic reduction Cr(VI) mechanism of Bi_2_WO_6_/Bi_2_S_3_/MoS_2_ under vis-light conditions.

We can now tentatively explain the mechanism underlying heterojunctions in photocatalytic reactions. When n-type Bi_2_WO_6_ is coupled with p-type Bi_2_S_3_ and MoS_2_, the n-p heterojunction is formed among semiconductors. The formation of the n-p heterojunction results in the equalization of their Fermi levels. This effect, in turn, induces band bending and a strong electric field at their interface. In this case, electrons and holes are prevented from coming into contact with each other due to the built-in electric field [74]. Meanwhile, according to the energy band structure, a type-I straddling heterojunction forms on the interface of MoS_2_ and Bi_2_S_3_, whereas a traditional type-II staggered heterojunction forms on the interface of Bi_2_S_3_ and Bi_2_WO_6_. MoS_2_ and Bi_2_S_3_ are excited when type-I MoS_2_/Bi_2_S_3_ are exposed to vis-light. The electrons on the CB of MoS_2_ will quickly transfer onto that of Bi_2_S_3_, and the holes on the VB of the MoS_2_ simultaneously hop onto that of Bi_2_S_3_. If no measures are taken, the electrons and holes accumulate in the Bi_2_S_3_ semiconductor and recombine rapidly. Interestingly, the existence of type-II Bi_2_S_3_/Bi_2_WO_6_ enables the electrons on the CB of the p-type Bi_2_S_3_ semiconductor to transfer directly onto the CB of n-type Bi_2_WO_6_, and the holes on the VB of Bi_2_WO_6_ can be spontaneously injected into the VB of Bi_2_S_3_. This phenomenon realizes the effective separation and transfer of photogenerated electron-hole pairs. The strong electric field generated by the n-p heterojunction further promotes this process. Compared with a single heterostructure, this system can better realize the separation and transfer of photogenerated electron–hole pairs under the joint action of multiple heterostructures. Given the above analysis, the proposed photocatalytic reduction mechanism of Cr(VI) by Bi_2_WO_6_/Bi_2_S_3_/MoS_2_ photocatalysis under vis-light is obtained (Scheme 2). When vis-light is irradiated on the Bi_2_WO_6_/Bi_2_S_3_/MoS_2_ heterojunction, electrons on the semiconductor VB are excited to CB and the corresponding numbers of holes are retained on the VB, thus forming photogenerated electron–hole pairs (Equations (8)–(10)). Electrons on the CB of type-*p* Bi_2_S_3_ and MoS_2_ are transferred to CB of type-*n* Bi_2_WO_6_, which is finally used for Cr(VI) reduction, whereas the holes remain in the VB of Bi_2_S_3_ (Equation (11)). Moreover, the CB edges of Bi_2_WO_6_, Bi_2_S_3_, and MoS_2_ are more negative than the reduction potential of *E*_(Cr(VI)/Cr(III))_ (0.51 eV) [61,67]. Theoretically, the reduction of Cr(VI) to Cr(III) can be feasibly achieved by this route. Eventually, the electrons in the system will reduce Cr_2_O_7_^2−^ to Cr(III), and the holes will oxidize H_2_O to produce O_2_ (Equations (12) and (13), respectively).

(8) $${\mathrm{Bi}}_{2}{\mathrm{WO}}_{6}\;+\;hv\;\to {\;e}^{-}{\mathrm{CB}\;+\;h}^{+}\mathrm{VB},$$

(9) $${\mathrm{Bi}}_{2}S_{3}\;+\;hv\;\to {\;e}^{-}{\mathrm{CB}\;+\;h}^{+}\mathrm{VB},$$

(10) $${\mathrm{MoS}}_{2}\;+\;hv\;\to {\;e}^{-}{\mathrm{CB}\;+\;h}^{+}\mathrm{VB},$$

(11) $${\mathrm{Bi}}_{2}{\mathrm{WO}}_{6}{(e}^{-}\mathrm{CB})\;-{\;\mathrm{Bi}}_{2}S_{3}{(e}^{-}\mathrm{CB})\;-{\;\mathrm{MoS}}_{2}{(e}^{-}{\mathrm{CB})\;+\;\mathrm{Bi}}_{2}{\mathrm{WO}}_{6}{(h}^{+}\mathrm{VB})\;-{\;\mathrm{Bi}}_{2}S_{3}{(h}^{+}\mathrm{VB})\;-{\;\mathrm{MoS}}_{2}{(h}^{+}\mathrm{VB})\;\;\to {\;\mathrm{Bi}}_{2}{\mathrm{WO}}_{6}{(}^{\mathrm{total}}e^{-}{\mathrm{CB})\;+\;\mathrm{Bi}}_{2}S_{3}{(}^{\mathrm{total}}h^{+}\mathrm{VB})\;,$$

(12) $${\mathrm{Cr}}_{2}O_{7}^{2-}{\;+\;14H}^{+}{\;+\;6e}^{-}\;\to {\;2\mathrm{Cr}}^{3+}{\;+\;7H}_{2}O,$$

(13) $${2H}_{2}{O\;+\;4h}^{+}\;\to {\;O}_{2}{\;+\;4H}^{+},$$

## 4. Conclusions

In summary, spherical Bi_2_WO_6_/Bi_2_S_3_/MoS_2_ n-p heterojunction ternary composites with vis-light response are prepared by a hydrothermal method. The HRTEM and Mott–Schottky curves confirm the formation of the n-p heterojunction. The XPS spectra show the existence of strong interaction and charge transfer among Bi_2_WO_6_, Bi_2_S_3_, and MoS_2_ in the n-p heterojunction. The effects of various factors on the catalytic activity of Bi_2_WO_6_/Bi_2_S_3_/MoS_2_ photocatalysts are investigated. For vis-light photocatalytic reduction of Cr(VI), the composites show higher photocatalytic reduction capacity than pure Bi_2_WO_6_, where BBM-3 exhibits the highest photocatalytic activity with a corresponding Cr(VI) reduction rate of up to 100% within 75 min. After three cycles of experiments, XRD and XPS analyses verify that the heterojunction possesses structural stability and can effectively reduce Cr(VI) into Cr(III). The improvement of photocatalytic activity of composite materials mainly benefits from the following points: First, the successful construction of a heterojunction structure forms a good interface contact, which promotes the effective separation of photogenerated electrons and holes. Second, the effective assembly and interfacial synergy between the three components enhance the vis-light absorption capacity of the samples and expand their light absorption range. Third, the increased surface area and abundant mesoporous structure endow the composites with more reactive sites and strong adsorption capacity of pollutants. The successful construction and application of Bi_2_WO_6_/Bi_2_S_3_/MoS_2_ n-p heterojunction in this work provide new ideas and strategies for the development of photocatalysts for wastewater treatment.

## Figures and Tables

**Table 1:** Summary of the band gap energy (*E*_g_), conduction band edge (*E*_CB_), and valence band edge (*E*_VB_) of Bi_2_WO_6_, Bi_2_S_3_, and MoS_2_.

Materials	*E*_g_ (eV)	*X* (eV)	*E*_e_ (eV)	*E*_CB_ vs. NHE ^1^ (eV)	*E*_VB_ vs. NHE ^1^ (eV)
Bi_2_WO_6_	2.74	6.20	4.50	0.33	3.07
Bi_2_S_3_	1.19 [51]	5.27	4.50	0.18	1.37
MoS_2_	1.30	5.32	4.50	0.17	1.47

^1^ NHE: normal hydrogen electrode.

**Table 2:** Performance comparison with other materials used for vis-light catalytic reduction of Cr(VI).

Materials/Amount (mg)	Cr(VI) Solution Volume (mL)/Concentration (mg L^−1^)	Time (min)	Photocatalytic Removal Rate	Publication Date	Ref.
CoS_2_/g-C_3_N_4_-rGO/10	20/20	120	99.8	2020	[63]
MoSe_2_/ZnO/ZnSe/80	80/20	180	100	2020	[67]
rGO/ZnO/Au/50	50/10	40	97	2020	[68]
Bi_2_MoO_6_/ZnO/100	50/50	150	100	2019	[61]
RGO/BiOI/ZnO/100	150/10	180	92	2019	[69]
Bi_2_WO_6_+Oxalic/60	50/10	120	100	2018	[70]
CuInS_2_ QDs/Bi_2_WO_6_/20	40/10	300	90	2018	[71]
Bi_2_WO_6_/MoS_2_/RGO+Lactic acid/30	100/10	80	100	2016	[72]
Bi_2_WO_6_/Bi_2_S_3_/MoS_2_/20	50/40	75	100		This work

## Supplementary Materials

The following are available online at https://www.mdpi.com/2079-4991/10/9/1813/s1: 1. Determination of Cr(VI) concentration using the DPC method; 2. Characterization data. Figure S1: High-magnification TEM image of BBM-3, Figure S2: EDS analysis of the BBM-3, Figure S3: XPS spectra of Bi_2_WO_6_, Bi_2_S_3_, MoS_2_, and BBM-3: (a) survey, (b) Mo 3d, (c) Bi 4f, and (d) W 4f spectra, Figure S4: N_2_ adsorption–desorption isotherms of the Bi_2_WO_6_, BBM-1, BBM-2, BBM-3, and BBM-4, Figure S5: The Mott–Schottky curves of (a) Bi_2_WO_6_, (b) Bi_2_S_3_, (c) MoS_2_, and (d) BBM-3, Figure S6: Photocurrent responses of Bi_2_WO_6_, BBM-1, BBM-2, BBM-3, and BBM-4, Figure S7: The UV–vis absorption spectra of Cr(VI) solution over the BBM-3 at (a) pH 4.00, and (b) pH 8.00, Figure S8: The UV–vis absorption spectra of Cr(VI) solution over the BBM-3 in the presence of (a) hole scavenger (citric acid) and (b) electron scavenger (KBrO_3_), Figure S9: The UV–vis absorption spectra of Cr(VI) solution over the BBM-3 in the cycle experiments: (a) 2nd run and (b) 3rd run.
