# Supplementary material for: Spherical Bi2WO6/Bi2S3/MoS2 n-p Heterojunction with Excellent Visible-Light Photocatalytic Reduction Cr(VI) Activity
Source: Nanomaterials (Basel). 2020 Sep 11;10(9):1813. doi: 10.3390/nano10091813 (PMC7558576; doi:10.3390/nano10091813)
Supplement: Supplementary file 1 [file nanomaterials-10-01813-s001.pdf]

1. Determination of Cr(VI) concentration using the DPC method

The concentration of Cr(VI) was measured colorimetrically at λ_{max} = 540 nm by the standard diphenylcarbazide (DPC) method. In detail, the filtered sample 1 mL was injected into a 50 mL volumetric flask and diluted to scale with water. Subsequently, 0.5 mL of sulfuric acid, phosphoric acid solution (acid/water volume ratio 1:1) and 2 mL of freshly prepared DPC solution were successively added to the mixture. After vortexing the mixture for about 1 min, it was allowed to stand for another 10 min in order to ensure full color development. Using the distilled water as reference, the absorbance of the colored Cr(VI)-DPC complex solution was then measured at λ_{max} = 540 nm.

Preparation of DPC solution: 100 mg of DPC was dissolved in 50 mL mixed solution of acetone and water (Volume ratio 1:1) in a 50 mL volumetric flask, shaken well and stored it in a brown reagent bottle.

2. Characterization data

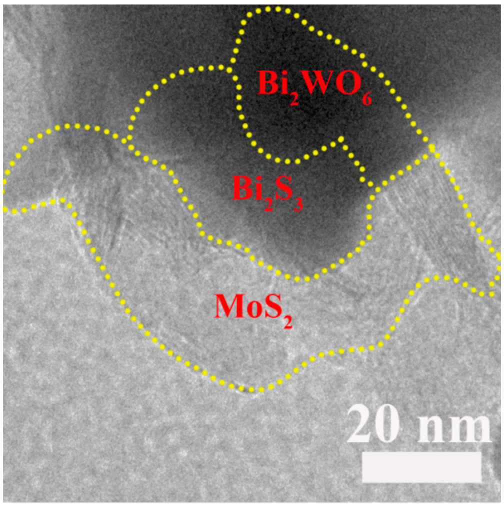

Figure S1. High-magnification TEM image of BBM-3.

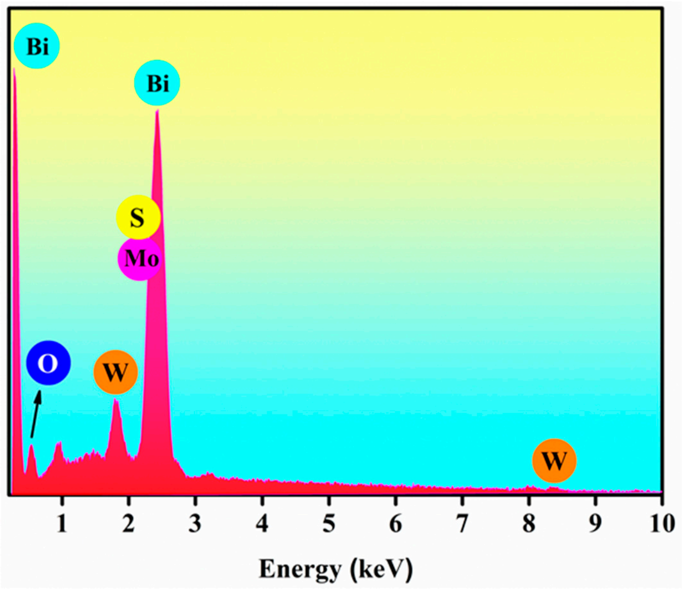

Figure S2. EDS analysis of the BBM-3.

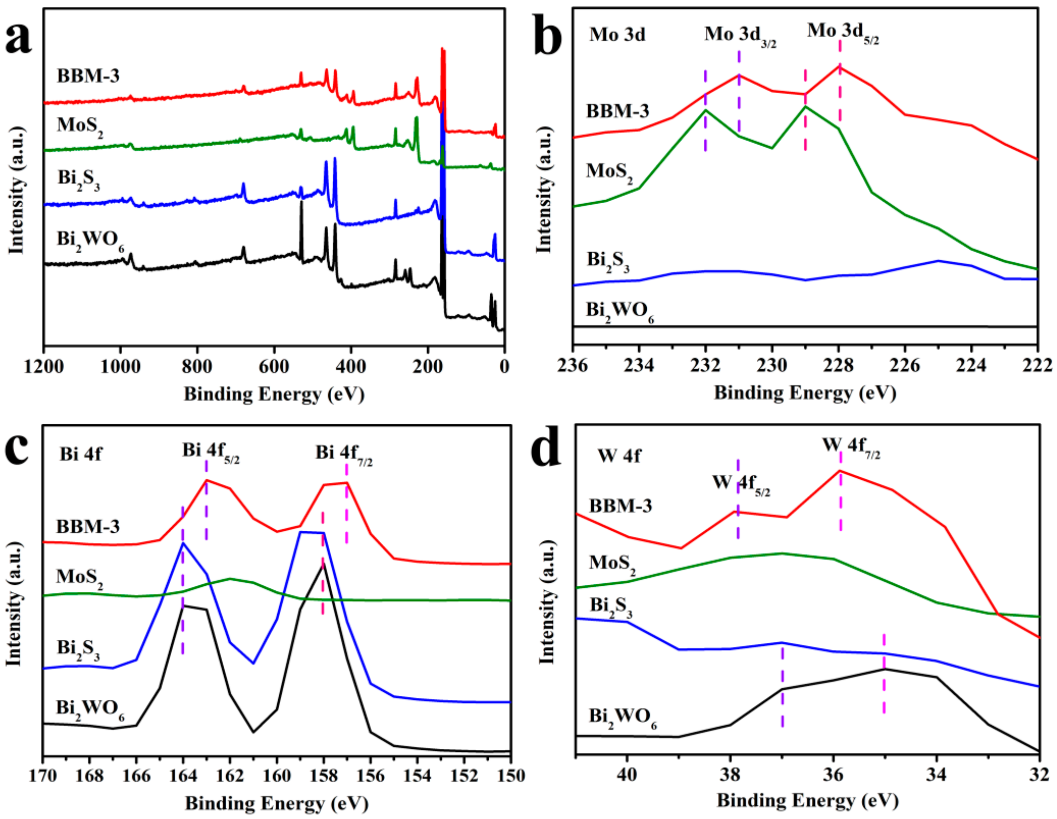

Figure S3. XPS spectra of Bi_2WO_6, Bi_2S_3, MoS_2, and BBM-3: (**a**) survey, (**b**) Mo 3d, (**c**) Bi 4f, and (**d**) W 4f.

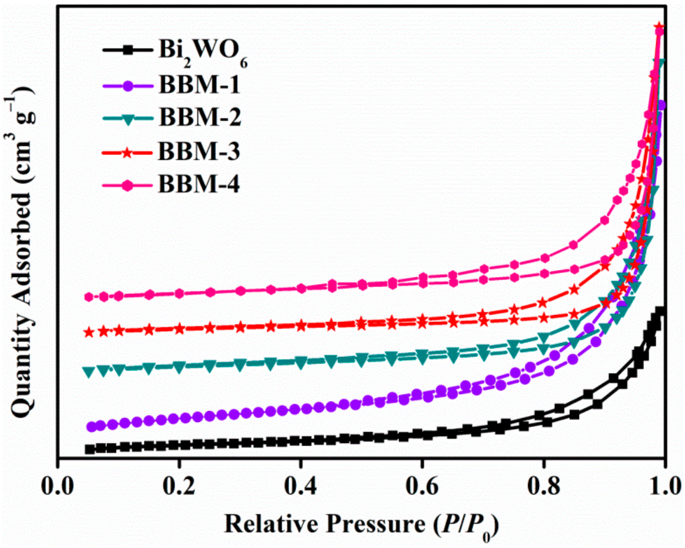

Figure S4. N_2 adsorption–desorption isotherms of the Bi_2WO_6, BBM-1, BBM-2, BBM-3, and BBM-4.

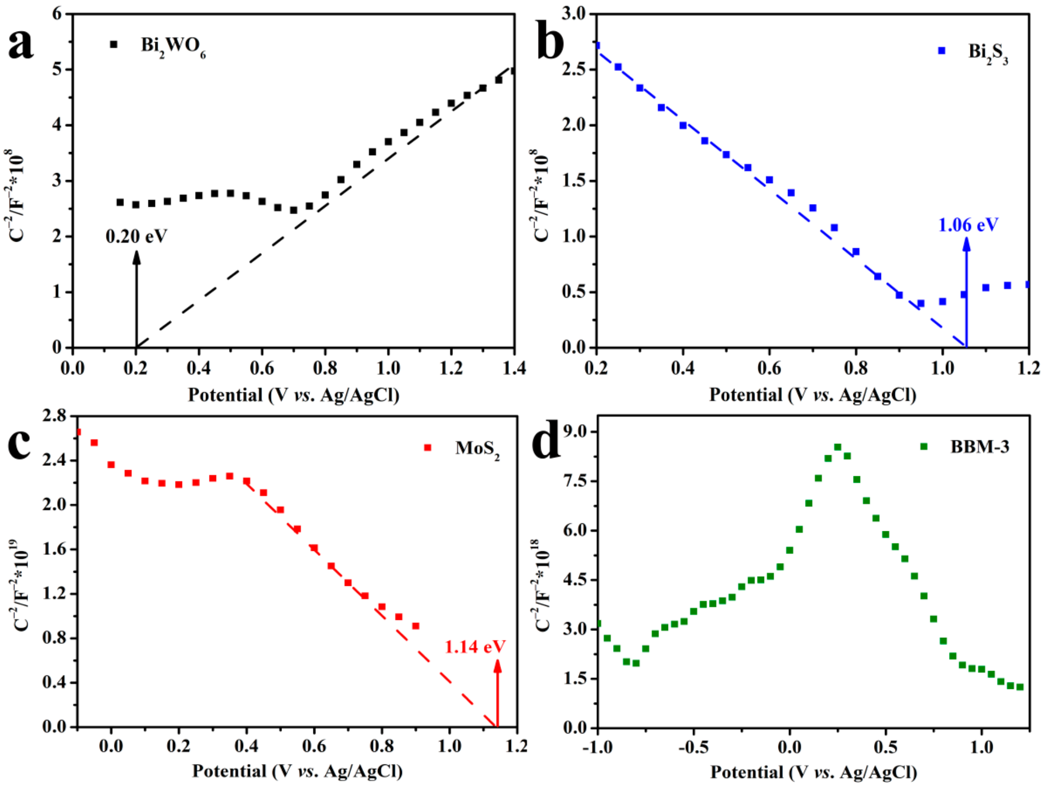

Figure S5. The Mott–Schottky curves of (**a**) Bi_2WO_6, (**b**) Bi_2S_3, (**c**) MoS_2, and (**d**) BBM-3.

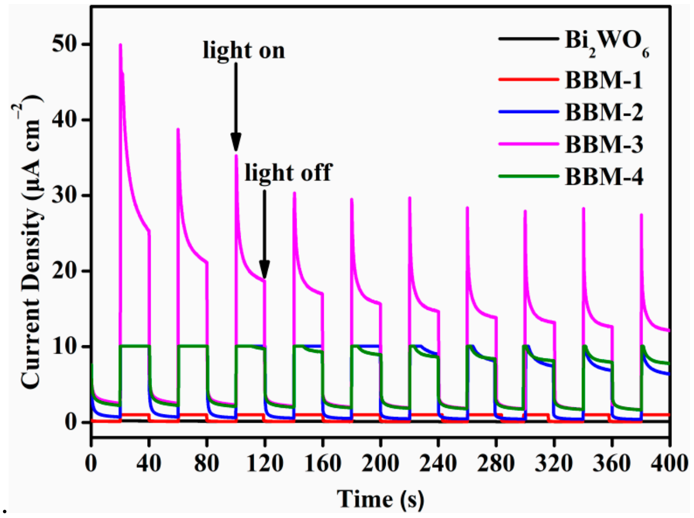

Figure S6. Photocurrent responses of Bi_2WO_6, BBM-1, BBM-2, BBM-3, and BBM-4.

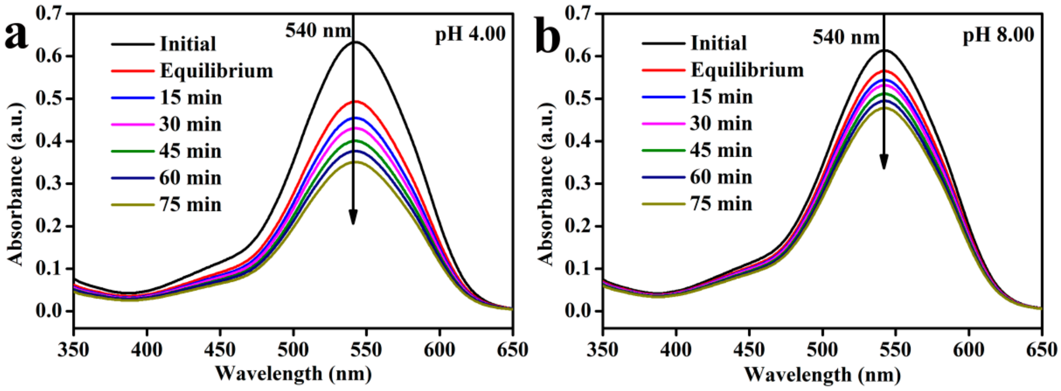

Figure S7. The UV–vis absorption spectra of Cr(VI) solution over the BBM-3 at (**a**) pH 4.00, and (**b**) pH 8.00.

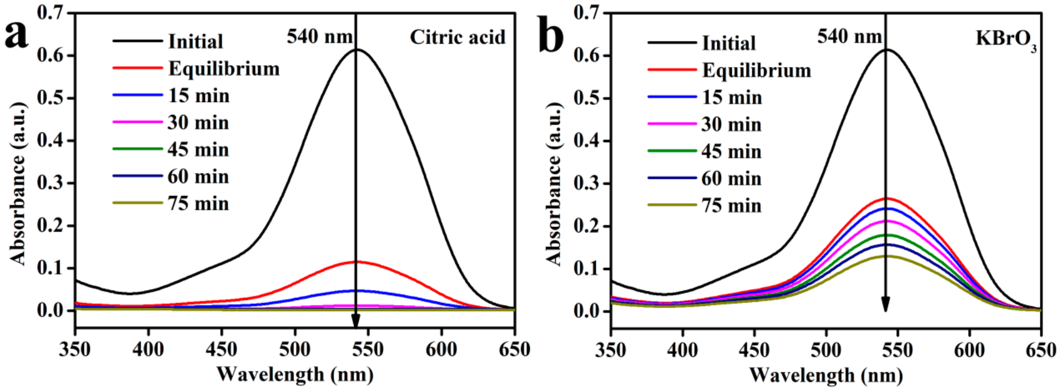

Figure S8. The UV–vis absorption spectra of Cr(VI) solution over the BBM-3 in the presence of (**a**) hole scavenger (citric acid) and (**b**) electron scavenger ($KBrO_3$).

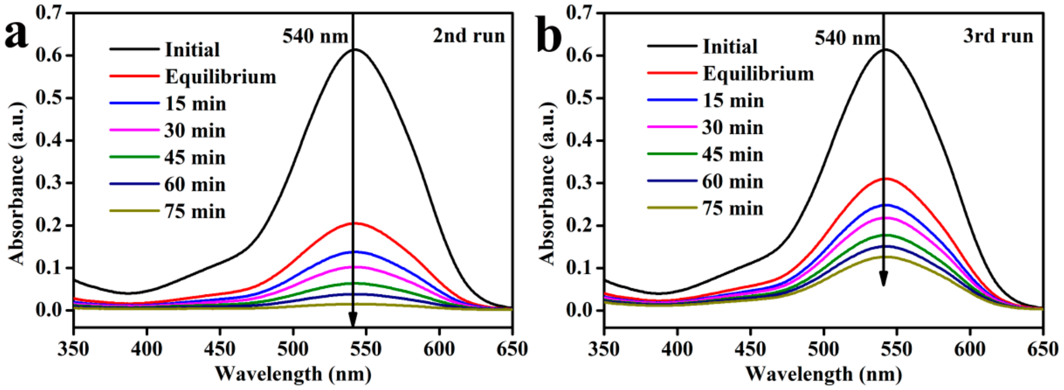

Figure S9. The UV–vis absorption spectra of Cr(VI) solution over the BBM-3 in the cycle experiments: (**a**) 2nd run and (**b**) 3rd run.
